# Male-Dependent Promotion of Colitis in 129 *Rag2^−/−^* Mice Co-Infected with *Helicobacter pylori* and *Helicobacter hepaticus*

**DOI:** 10.3390/ijms21238886

**Published:** 2020-11-24

**Authors:** Zhongming Ge, Lili Ge, Sureshkumar Muthupalani, Yan Feng, James G. Fox

**Affiliations:** Division of Comparative Medicine, Massachusetts Institute of Technology, Cambridge, MA 02139, USA; lge12345@gmail.com (L.G.); smuthu01@mit.edu (S.M.); yanf@mit.edu (Y.F.)

**Keywords:** *H. pylori*, *H. hepaticus*, co-infection, colitis promotion, inflammatory responses, 129 *Rag* mice

## Abstract

The prevalence of gastric *Helicobacter pylori* (Hp) infection is ~50% of the world population. However, how Hp infection influences inflammatory bowel disease in humans is not fully defined. In this study, we examined whether co-infection with Hp influenced *Helicobacter hepaticus* (Hh)–induced intestinal pathology in *Rag2^−/−^* mice. *Rag2^−/−^* mice of both sexes were infected with Hh, of which a subgroup was followed by infection with Hp two weeks later. Co-infected males, but not females, had significantly higher total colitis index scores in the colon at both 10 and 21 weeks post-Hh infection (WPI) and developed more severe dysplasia at 21 WPI compared with mono-Hh males. There were no significant differences in colonization levels of gastric Hp and colonic Hh between sexes or time-points. In addition, mRNA levels of colonic *Il-1β*, *Ifnγ*, *Tnfα*, *Il-17A*, *Il-17F*, *Il-18*, and *Il-23*, which play important roles in the development and function of proinflammatory innate lymphoid cell groups 1 and 3, were significantly up-regulated in the dually infected males compared with mono-Hh males at 21 WPI. These data suggest that concomitant Hp infection enhances the inflammatory responses in the colon of-Hh-infected *Rag2^−/−^* males, which results in more severe colitis and dysplasia.

## 1. Introduction

*Helicobacter pylori* (Hp) is an important human pathogen that colonizes the stomach. Despite vigorous efforts to eradicate this bacterium with antibiotic treatments for the past 3 decades, the prevalence of Hp remains high; approximately 17% in developed countries to 85% in some developing countries [1]. Hp infection causes chronic active gastritis, leads to peptic ulcer diseases in some patients, and is a major risk factor for the development of gastric adenocarcinoma [2]. In addition, epidemiological findings indicate that Hp infection is associated with the increased risk of the development of colorectal cancer (CRC) [3]. Interestingly, it was reported that seropositivity of Hp VacA, a known virulence factor, was associated with increased risk of CRC among African Americans but not among white populations in the United States [4]. In contrast, some epidemiological data suggest that Hp infection is protective in the development of inflammatory bowel disease (IBD) in humans [5]. Such associations were further supported by murine models of gastric Hp infection in which prior Hp infection in female C57BL/6 mice attenuated *Salmonella typhimurium* enteritis or dextran sodium sulphate-induced colitis [6,7]. Effects of Hp infection and the underlying mechanisms on the development of IBD and CRC still remains unclear.

The genus *Helicobacter* consists of over 40 named species (http://www.ncbi.nlm.nih.gov/Taxonomy/Browser/helicobacter), many of which have pathogenic potential, and have a wide spectrum of hosts [8]. Members of this genus have been increasing rapidly, and are divided into gastric and enterohepatic *Helicobacter* spp. (EHS) based on to their primary colonization niches [8]. Hp is the prototype of gastric helicobacters, whereas *Helicobacter hepaticus* (Hh) is the prototype of EHS [8,9]. The presence of the EHS 16S rRNA genes noted by PCR assays, including Hh in human subjects, has also been reported [8,10]. Hh is a murine pathogen which causes hepatitis, liver cancer, inflammatory bowel disease, and intestinal carcinoma in susceptible mouse strains [9,11]. Rodent models of Hh infection, particularly in 129 *Rag*-deficient mice, have been widely utilized to investigate the pathogenesis of infectious IBD and colitis-associated carcinogenesis in humans [11,12,13,14]. Our previous study demonstrated that concurrent infection of two EHS species *H. bilis* and *H. muridarum* attenuated Hp gastric pathology in female C57BL/6 mice [15]. In contrast, co-infection with Hh promoted Hp-induced gastric disease at 6 months post-Hp inoculation (MPI) but not at 11 MPI which was correlated with enhanced transcription of gastric *Il-17A* [16]. Il-17A, a marker for both proinflammatory Th17 cells and innate lymphoid cell (ILC) group 3, plays an important role in the development of Hh-induced colitis in 129SvEv *Rag^−/−^* mice [14]. We hypothesized that passage of Hp from the stomach into the lower bowel would influence mucosal immune responses, including ILCs. In this study, we investigated how Hp infection influenced Hh induced, innate immunity-driven intestinal carcinogenesis in 129S6/SvEvTac-*Rag2^tm1Fwa^* (RAG2) mice deficient in adaptive immunity despite the fact that mono-Hp infection does not cause gastrointestinal pathology in this immune-deficient mouse model [17].

## 2. Results

### 2.1. Co-Infection with Hp Promoted Male-Dependent Hh–Induced Colonic Premalignant Lesions

**Figure 1:**
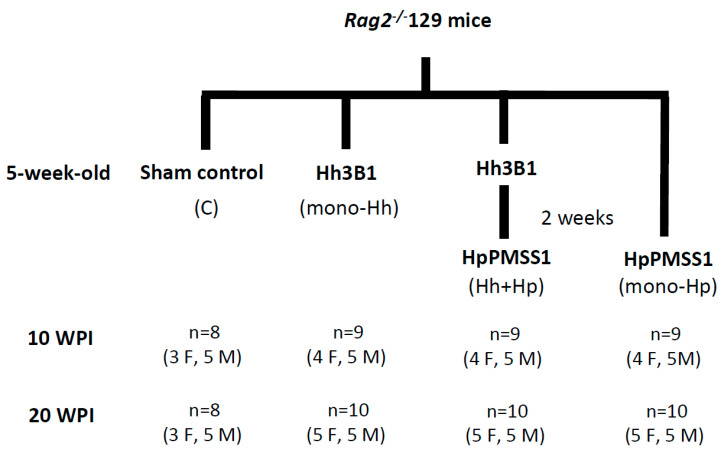
Schematic depiction of experimental design of infection. Five-week-old mice were inoculated with Hh 3B1; a subset of Hh -infected mice was then inoculated with Hp PMSS1 two weeks later. Abbreviations: Hh3B1 for *H. hepaticus* 3B1; HpPMSS1 for *H. pylori* PMSS1; M for male; F for female.

We previously demonstrated that concurrent infection of Hh promoted Hp-induced gastric disease at 6 months post-Hp inoculation in female C57BL/6 mice, whereas co-infection with other two EHS species *H. bilis* and *H. muridarum* attenuated Hp gastric pathology [15,16]. In this study, we tested whether co-infection with gastric Hp affected Hh-induced intestinal carcinogenesis in RAG2 mice. The experimental design was schematically presented in Figure 1. Infection with Hp in these mice with or without Hh did not induce any significant gastritis or other pathological alterations in the stomach (Figure S1), consistent with previous reports [17]. Hh infection, regardless of Hp infection status, did not elicit overt gastric pathology (Figure S1). Additionally, mono-Hp infected mice, similar to the sham controls, did not develop overt cecal and colonic pathology other than altered mucosa-associated lymphoid tissue configuration (lacking typical B and T cell appearance, germinal centers) and infrequent sparse neutrophilic and histiocytic infiltrates in a few animals (Figures S2 and S3).

**Figure 2:**
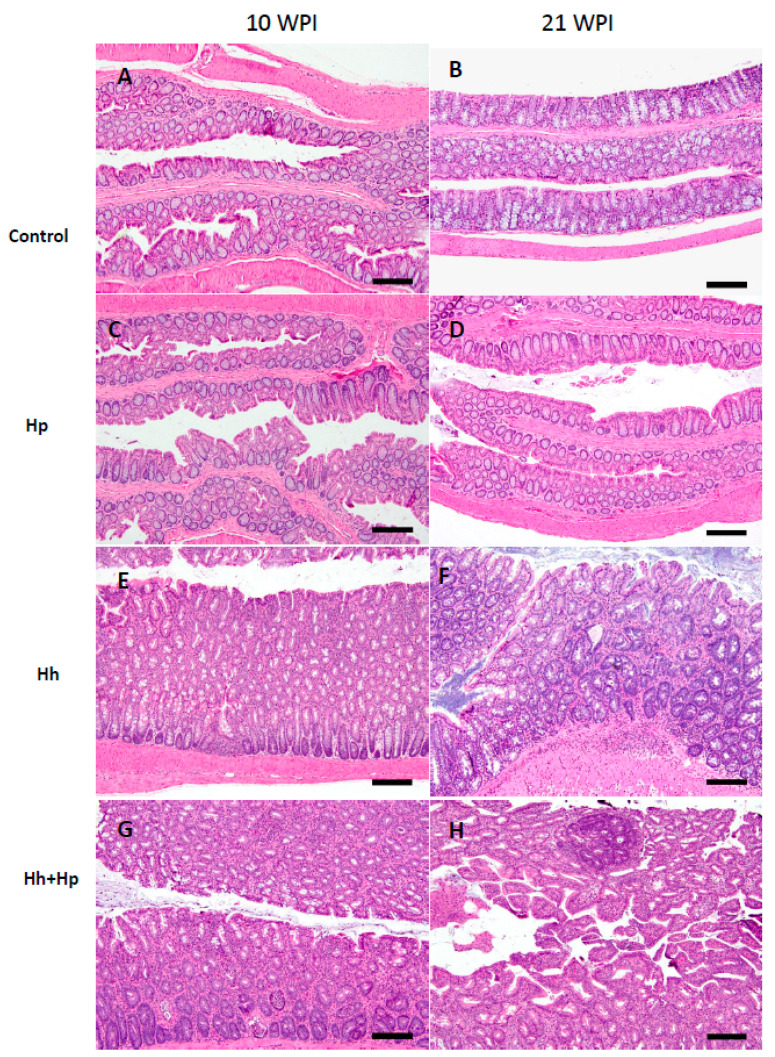
Representative H&E images of colon from male mice of different groups at 10 WPI and 21 WPI. (**A**): uninfected male, 10 WPI. (**B**): uninfected male at 21WPI. (**C**): mono-Hp male at 10 WPI. (**D**): Hp infected male at 21WPI. Panels a-d show none (a,b) to sparse (c&d) lamina propria inflammatory aggregates. (**E**): Hh infected male at 10 WPI showing moderate diffuse mucosal inflammation with hyperplastic and mildly ectatic glands and mild dysplasia. (**F**): mono-Hh male at 21 WPI showing moderate to severe mucosal and submucosal inflammation with ectatic and hyperplastic glands and moderate dysplasia. (**G**): Hh+Hp male at 10 WPI showing moderate to severe mucosal inflammation with crypt abscessation, ectatic glands, surface epithelial tethering, and hyperplastic and dysplastic glands. (**H**): Hh+Hp male at 21 WPI showing diffuse mucosal inflammation and villo-papillary hyperplastic and adenomatous epithelial proliferation. BAR, all images: 150 µM.

**Figure 3:**
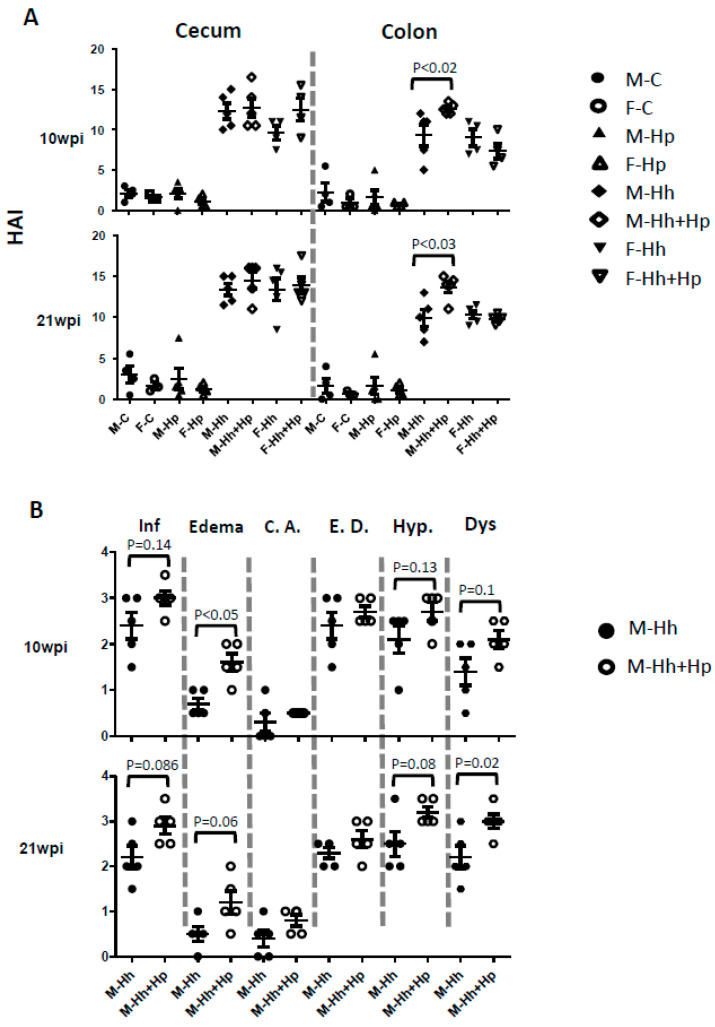
Co-infection with Hp promoted Hh-induced colonic lesions compared with mono- Hh in a male-dependent manner. Cecum and colon from RAG mice infected with Hh, Hp or co-infected with Hh+Hp for 10 to 21 weeks (WPI) (*n* = 3–5 per group) were graded for inflammation (Inf), edema, crypt atrophy (C.A.), epithelial defects (E.D.), hyperplasia (Hyp.) and dysplasia (Dys.). (**A**) Intestinal histologic activity index (HAI) was generated by combining scores for all sub-categorical lesions. (**B**) Scores of sub-categorical lesions of colon in male mice.

All Hh-infected mice, regardless of Hp infection status or sex, developed significantly severe cecal and colonic pathology compared to the sham controls and mono-Hp groups (Figure 2 and Figure 3A, *p* < 0.0001). The lesions in the cecum and colon were characterized by moderate to severe mucosal and submucosal inflammation comprising predominantly of dense coalescing aggregates of granulocytes (neutrophils, eosinophils) and macrophages intermingled with few mononuclear cells with lymphoid phenotypic appearance that were interpreted as innate lymphoid cells as mature lymphocytes are typically absent in RAG2 mice. The lesions were slightly more severe in the cecum compared to colon (Figure 2 and Figure 3, Figures S2 and S3) and were also slightly more severe at 21 WPI (weeks post-Hh infection) than 10 WPI. In addition to inflammation, there were prominent epithelial defects characterized by surface erosions, epithelial degeneration/necrosis, ectactic glands, and crypt abscesstation as well as edema, mild crypt atrophy, marked epithelial hyperplasia, and moderate to severe dysplasia, which, in a few animals, progressed to villo-papillary adenomatous proliferations (intra-epithelial neoplasia) and/or invasive carcinoma (intramucosal or submucosally invasive carcinoma). In the colon, the lesions were observed in the distal colon, rectum, mid or transverse colon, and proximal colon with a decreasing order of severity. Cecal lesions were more severe at the ileo-cecal junction and in most instances also extended to the entire cecum in varying grades.

There were no significant differences in cecal and colonic HAI (Histologic Activity Index) scores between mono-Hh and Hh+Hp females at both time-points (Figure 3A, Figures S2 and S3). However, the Hh+Hp males had comparable cecal HAI scores, but had significantly higher colonic pathology HAI scores compared to mono-Hh males at both time-points (Figure 3A, *p* < 0.05). For sub-categorical colonic lesions including inflammation, edema, crypt atrophy, epithelial defect, hyperplasia, and dysplasia, Hh+Hp males developed only more severe edema at 10 WPI compared with mono-Hh mice (Figure 3B, *p* < 0.05). At 21 WPI, colonic dysplasia was significantly more severe in Hh+Hp males compared to mono-Hh males (Figure 3B, *p* = 0.02); inflammation (*p* = 0.086), edema (*p* = 0.06) and hyperplasia (*p* = 0.08) in Hh+Hp males tended to be more severe than those in mono-Hh males. Additionally, dysplasia (*p* < 0.02) and hyperplasia (Figure 3B, *p* = 0.076) were more severe in the colon of Hh+Hp males at 21 WPI compared to 10 WPI; there were no significant differences in severity of colonic dysplasia and hyperplasia in mono-Hh females between these two time-points (Figure 3B, *p* > 0.1). These results indicate that co-infection with Hp significantly promoted progression of Hh-induced colonic carcinogenesis in RAG2 males.

### 2.2. Males Co-Infected with Hp Produced Higher mRNA Levels of Colonic Cytokines Involved in the Development and Function of Innate Lymphoid Cells 1 and 3

**Figure 4:**
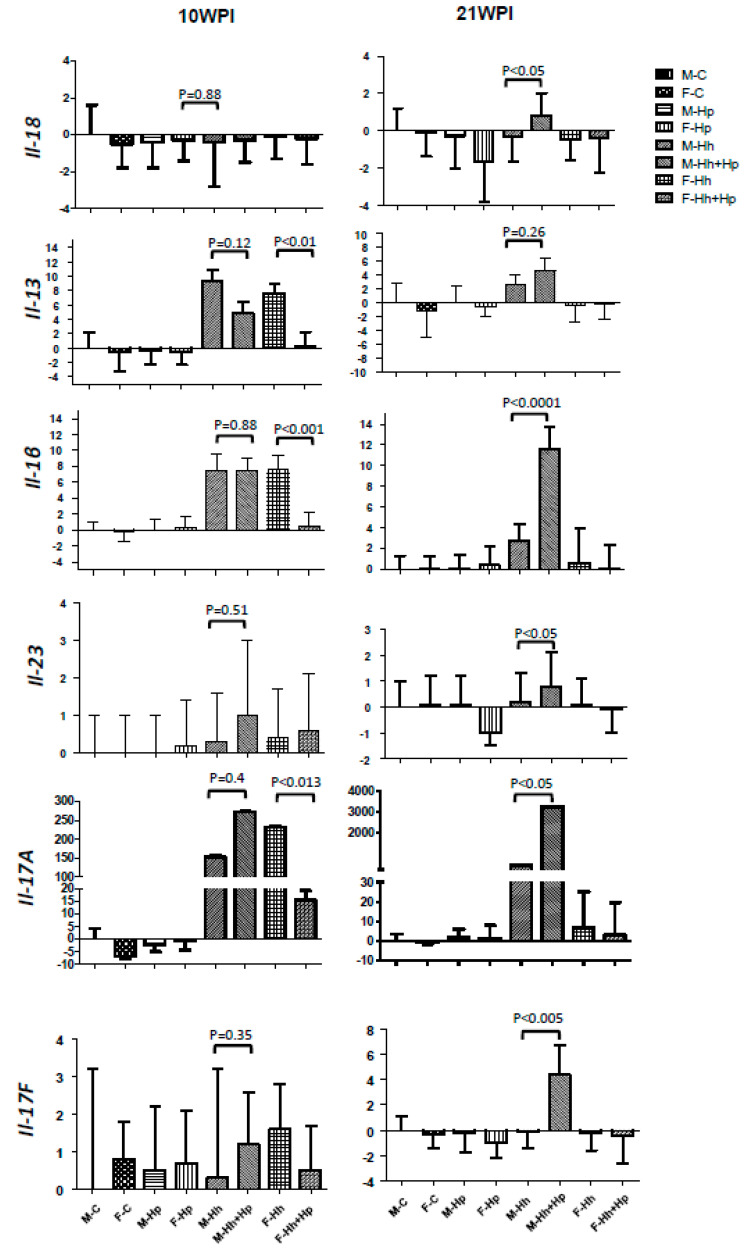

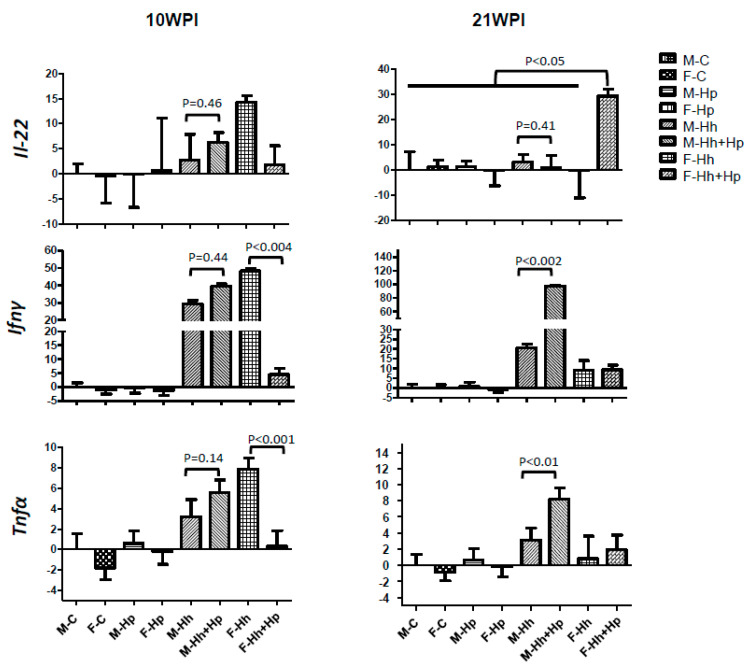
Co-infection with Hp in males increased mRNA levels of colonic ILC1s- and ILC3-associated cytokines compared to mono-Hh males at 21 WPI. Total RNA prepared from colonic tissues of mice infected or sham-dosed were evaluated by qPCR for expression levels of mRNA for select cytokines, which then were normalized to the expression of the house-keeping gene *Gapdh*. The Y axis represents the mean fold change (±standard deviation) of the mRNA levels in reference to uninfected male controls.

We previously reported that female C57BL/6 mice co-infected with Hh promoted Hp gastric pathology in concert with significant up-regulation of gastric *Il-17A* at 6 months post inoculation [16]. In addition, it has been documented that intestinal Il-1β and Il-17A produced by ILCs play an important role in the development of Hh-induced colitis in RAG2 mice [14]. Next, we examined mRNA expression in the cecum and colon of selected cytokines involved in intestinal carcinogenesis and functions of ILCs. In the colon, there were no significant differences in mRNA levels of *Il-18*, an ILC1-associated cytokine [18], among all groups at 10 WPI, whereas Hh+Hp males produced significantly more *Il-18* transcripts compared to mono-Hh males at 21 WPI (Figure 4, *p* < 0.05). The mRNA levels of a ILC2-associated colonic cytokine Il-13 were comparable between mono-Hh and Hh+Hp males at both time-points; mono-Hp females, mono-Hh and Hh+Hp males had higher expression of colonic *Il-13* mRNA compared to the remaining groups including Hh+Hp females (Figure 4, *p* < 0.014). The mRNA levels of colonic ILC3/natural killer (NK) cells -associated cytokines, including *Il-1β*, *Il-23a*, *Il-17A* and *Il-17F* were significantly higher in Hh+Hp males compared to mono-Hh males at 21 WPI (Figure 4, *p* < 0.05 to 0.001). However, there was no significant difference in mRNA levels of the ILC3-associated cytokine *Il-22* between Hh+Hp and mono-Hh males (Figure 4, *p* > 0.4). In addition, when compared to mono-Hh males, Hh+Hp males contained significantly higher mRNA levels of colonic *Ifnγ* and *Tnfα* at 21 WPI, both of which are illustrative of the important roles for the function of ILC1s and ILC3s. Interestingly, mono-Hh females had significantly higher mRNA levels of colonic *Il-1β*, *Il-17A*, *Ifnγ*, *Tnfα*, and *Il-13* than Hh+Hp females (Figure 4, *p* < 0.01); at 21 WPI Hh+Hp females had significantly higher mRNA levels of colonic *Il-22* when compared to the remaining groups (Figure 4, *p* < 0.05).

To investigate if other proinflammatory or pro-oncogenic genes play a role in the promotion of Hh-induced colonic cancer by Hp in a male-dependent manner, colonic genes including *iNos*, *Il-6*, *Il-10*, and *regIIIγ* were examined. Hh+Hp males produced significantly higher mRNA levels of colonic *iNos* than mono-Hh males at 10 WPI (*p* < 0.05); this difference trended higher in Hh+Hp mice (*p* = 0.062) at 21 WPI (Figure S4). There were no significant differences in mRNA levels of colonic *regIIIγ*, *Il-6*, and *Il-10* between Hh+Hp and mono-Hh males (Figure S4). Collectively, these results indicate that co-infection with Hp in RAG2 males preferably enhanced gene expression of ILC1s and ILC3s-associated colonic cytokines. In contrast, this co-infection in RAG2 females downregulated expression of several cytokines such as *Il-1β*, *Il-17A*, *Ifnγ*, *Tnfα*, and *Il-13* only at 10 WPI, but increased transcription of colonic *Il-22* at 21 WPI.

Since there was no significant difference in promoting severity of cecal pathology by Hh+Hp infection, mRNA expression of selected key cecal cytokines including *Il-1β*, *Tnfα*, *Ifnγ*, *Il-17A*, *Il-13*, *iNos*, *Il-6*, and *Il-10* were examined (Figure S5). Hh+Hp males had significantly higher mRNA levels of cecal *Il-17A* and *iNos* compared to mono-Hh males at 10 WPI but not at 21 WPI, whereas there were no significant transcriptional differences for the remaining gene targets (Figure S5). At 10 WPI, mRNA levels of cecal *Il-1β*, *Tnfα*, *Ifnγ*, *Il-17A*, *Il-13*, *iNos*, and *Il-6* were significantly higher in Hh-infected mice, irrespective of Hp infection or sex, except for *Il-13* in Hh+Hp females and *Il-6* in the mono-Hh females, when compared to the sham control or mono-Hp mice of both sexes (Figure S5, *p* < 0.05 or lower). The Hh+Hp females had significantly more expression of cecal *Il-13* compared to the sham controls, while cecal *Il-6* mRNA levels were comparable between the mono-Hh female and the sham controls or mono-Hp mice (Figure S5). At 21 WPI, Hh-infected mice contained higher mRNA levels of cecal *Ifnγ*, *Il-1β*, *Il-17A* and *iNos* compared to the sham controls or mono-Hp mice (Figure S5, *p* < 0.05 or lower). Interestingly, female controls produced significantly lower mRNA levels of cecal *Tnfα*, *Il-1β*, and *Ifnγ* compared to the male counterparts; mono-Hp males contained lower mRNA levels of cecal *Tnfα* compared to the sham males (Figure S5, *p* < 0.02 or lower). For cecal *Il-10* mRNA levels, Hh+Hp females contained significantly higher mRNA levels of cecal *Il-10* compared to the sham females (*p* < 0.005) or mono-Hh females (*p* < 0.003) at 10 WPI (Figure S5); there were significantly higher mRNA levels of cecal *Il-10* in mono-Hh females compared to the sham females at both time-points (Figure S5, *p* < 0.04).

### 2.3. Colonization Levels of Gastric Hp and Intestinal Hh Were Minimally Influenced by Co-Infection or Sex

**Figure 5:**
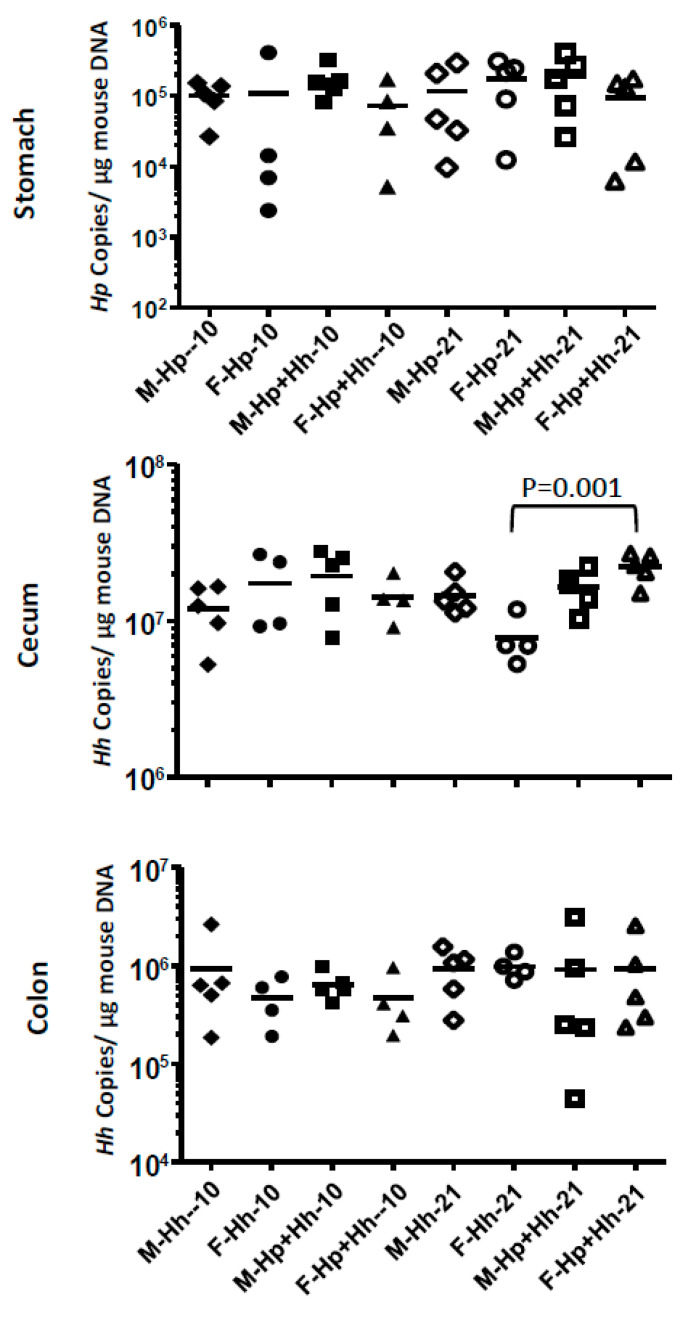
qPCR quantitation of gastric Hp and intestinal Hh. Copy numbers of gastric Hp PMSS1 or intestinal Hh genome are expressed per µg mouse DNA in the respective samples, determined using the 18S rRNA gene-based probe.

Colonization levels of gastric Hp or intestinal Hh were determined by qPCR. Average levels of Hp in stomach were approximately 1 × 10^5^ per µg mouse DNA (Figure 5). There was no statistical difference in gastric Hp levels between Hh+Hp and mono-Hp groups or sexes in the same group at both time-points. Average colonization levels of cecal Hh were ~10^7^ organisms per µg mouse DNA, which is about 10-fold higher compared to those in the colon. There was no statistical significance in cecal Hh levels between mono-Hh infected and Hh+Hp mice or sexes at 10 WPI. At 21 WPI, co-infected females had higher cecal Hh levels compared to mono-Hh females (Figure 5, *p* = 0.001). There was no statistical significance in Hh colonization levels between the sexes or infection status at either time point in the colon.

## 3. Discussion

Hp infection induces gastric disease in immune-competent mouse strains such as C57BL/6 and INS-GAS mice [15,19]. In contrast, no overt gastric pathology developed in Hp-infected immune-deficient mouse strains such as *SCID* or RAG2 mice lacking mature T and B lymphocytes, unless the mice were reconstituted with effector T cells [17,20]. In spite of the absence of Hp-induced gastric pathology in RAG2 mice, our data demonstrated that co-infection with Hp in Hh-infected RAG2 male mice significantly promoted Hh-induced preneoplastic lesions in the colon but not in the cecum by 21 WPI. The increased severity of colonic pathology in Hh+Hp male mice was not related to colonization levels of cecal or colonic Hh or levels of gastric Hp, but instead was associated with enhanced expression of the colonic cytokines including *Il-18*, *Il-23*, *Il-17*, *Ifnγ*, *Il-1β*, and *Tnfα* at 21 WPI, all of which play important roles in the development and function of proinflammatory ILCs 1/NK and 3 [18,21].

ILCs have been recently identified as a heterogeneous cell population that function as a distinct arm of the innate immunity system [22]. Increasing evidence indicates that these cells can orchestrate the host’s immunity to infection, regulate tissue homeostasis and also promote inflammation and tumor progression when ILCs responses are dysregulated [18,21,23]. Functionally, both ILC1s and ILC3s promote chronic inflammation via production of proinflammatory cytokines TNFα, Ifnγ, Il-17, and Il-22, whereas ILC2s, like Th2 cells, release anti-inflammatory cytokines such as Il-4, Il-5, and Il-13 [22]. Non-cytotoxic ILC1s, which are stimulated by the presence of Il-12 and Il-18, confer immunity to infection with intracellular bacteria and protozoa, while Il-1β/Il-23-dependent ILC3s response protects the host from infection with extracellular bacteria [18]. Prior studies reported that non-gastric *Helicobacter* species, including Hh, *H. typhlonius* and *H. apodemus*, promoted the host tolerance via induction of regulatory T cells that restrained Hh-specific proinflammatory Th17 cells in immunocompetent C57BL/6J mice, but induced colitogenic Th17 cells during colitis in immunocompromised mice (*Rag1^−/−^* and Il10-deficient B6 mice) [24,25]. In immune-deficient RAG2 mice, ILC3s were shown to play an important role in the development of Hh-induced colitis [14]. Enhanced secretion of Il-1β from colonic lamina propria leukocytes (cLPL) of Hh-infected RAG2 mice increased accumulation of Il-17A-expressing ILCs and also were associated with increased secretion of cLPL Ifnγ and Infα [14]. In our current study, more severe colonic pathology in the Hh+Hp males when compared to their mono-Hh counterparts was coupled with significant transcriptional up-regulation of colonic Il-18 (ILC1s) and Il23/Il-1β (IlC3s) but not Il-13 (ILC2s), indicating that co-Hp infection promotes male-dependent Hh induced colonic pathology, likely driven by enhancing the function of ILC1s and ILC3s [18]. Our hypothesis is further supported by the results that mRNA levels of *Tnfα* and *Ifnγ* produced by both ILC1s and ILC3s as well as Il-17A and Il-17F produced by ILC3s were significantly higher in Hh+Hp males than mono-Hh males at 21 WPI. Intriguingly, expression of colonic *Il-22* in the current study, an effector cytokine released by ILC3s, and its downstream gene *RegIIIγ* were comparable in colons between Hh+Hp and mono-Hh 129 *Rag2^−/−^* males. In another study, anti-Il-22 but not anti-Il-17 treatment significantly reduced the incidence rate of colorectal cancer in Hh+AOM-treated 129*Rag2^−/−^* mice [26]. This discrepancy between these two studies is likely due to differences in experimental design: dual helicobacter infection-induced colitis versus Hh+AOM-induced CRC. It is plausible to hypothesize that in our model Hp-promoted colitis in Hh-infected males could be mediated by enhancing the response of a CCR6+ T-bet- *Il17*-producing subset of ILC3s [18]. This hypothesis is mirrored by the promotion of Hp induced gastric pathology by co-infected Hh in C57BL/6 mice was positively correlated with enhanced expression of gastric *Il-17* [16]. However, further studies such as examination and comparison of the populations of the colonic ILCs between mono-Hh and Hh+Hp males and between Hh+Hp males and Hh+Hp females are required to define the role of intestinal ILCs 1 and 3 in Hp-associated promotion of Hh colitis. Additionally, *Rag2^−/−^Il2rg^−/−^* mice lacking all ILCs or *Rag2^−/−^Rorc(gt)^−/−^* mice lacking ILC3 can be utilized to dissect how intestinal ILCs or ILC 3 contribute to male-biased promotion of Hh colitis by Hp infection.

Up-regulation of colonic proinflammatory cytokines in Hh+Hp males compared to mono-Hh males became statistically significant at 21 WPI but not at 10 WPI. This result was consistent with progression of the major sub-categorical lesions of these infected mice during chronic infection. At 10 WPI, there were no significant differences in five of six sub-categorical features (except for edema) of colonic lesions between Hh+Hp and mono-Hh males in spite of higher HAI score (in aggregate of the sub-categorical lesions) in Hh+Hp males compared to mono-Hh males. These data indicate that Hh+Hp males, when compared to mono-Hh males, have greater pathogenic potential, but the associated transcriptional enhancement of the aforementioned proinflammatory cytokines was not sufficient to be statistically significant at 10 WPI. By 21 WPI, however, dysplasia was significantly more severe in Hh+Hp males compared to mono-Hh males, which was in concert with a significant increase of the colonic inflammatory responses. Given that Hp only colonized the stomach and didn’t induce gastric pathology with or without Hh coinfection, the mechanism underlying Hp-induced promotion of Hh colitis needs to be further delineated. Since the genomes of Hp and Hh share approximately 50% of their respective orthologs, we hypothesize that these two helicobacters share one or more factors that can stimulate inflammatory responses [27]. These unidentified factors from Hp may sensitize gastric macrophages or dendritic cells that subsequently migrate to the colonic tissue inflamed by Hh infection, thereby enhancing the inflammatory responses noted in our study. This notion is supported by the finding that the dendritic cells in the Peyer’s patches phagocytosed coccoid Hp in the small intestine of mice and subsequently primed T cells [28].

No impact of Hp coinfection on colonic pathology in RAG2 females is consistent with the lack of upregulation of colonic cytokines including *Il-1β*, *Il-17A*, *Ifnγ* and *Tnfα* in Hh+Hp females compared to mono-Hh females at 10 WPI, which play a pivotal role in inducing intestinal inflammation and carcinogenesis [12,13]. This male-biased promotion of Hh-induced colonic pathology caused by Hp coinfection is likely attributable to less susceptibility of RAG2 females versus males in response to Hh infection. This premise is supported by our previous work illustrating that Hh infection induced more invasive cancer in the lower bowel of Rag2-deficient male mice receiving Il10-deficient regulatory T cells compared to their female counterparts [29]. In addition, our recent study reported that Hh infection increased genomic mutation frequencies in male but not female Il10-deficient RAG2 *gpt* mice compared with their respective controls, suggesting that males are more susceptible to genomic mutations promoted by Hh infection [30].

Our present findings appear inconsistent with selected epidemiological data suggesting that Hp infection has an inverse relationship with the development of IBD, although some studies did not confirm this observation [5,31]. In a meta-analysis of the possible protective effects of Hp on IBD development, the authors noted that there was significant heterogeneity in the results and that the possibility of publication bias limit the validity of these conclusions [5]. For example, one study showed that Hp-negative gastritis was positively associated with IBD. However, Hp negativity was determined using a *Helicobacter*-specific immunohistochemical stain on gastric tissue and did not use other biomarkers of Hp infection [32]. Another gastric microbiome study illustrates the possibility that conventional Hp testing could lead to false negative Hp gastritis samples; only 11 (serum Hp IgG ELISA) or 12 (histopathology) of the 19 human gastric biopsies containing the Hp 16S rRNA gene sequence were *H. pylori*-positive [33].

Male-biased promotion of Hh colitis coinfected with Hp in RAG2 mice in the current study is not in agreement with previous findings on effect of Hp infection on bacterium- or chemically induced colitis in female C57BL/6 mice [6,7]. Prior infection with Hp or administration with Hp SS1 DNA ameliorated colitis induced by infection with *Salmonella typhimurium* or treatment with dextran sodium sulphate [6,7]. This effect was mediated by suppression of two proinflammatory cytokines Il-12 and type-1 Ifn produced by dendritic cells with Hp DNA that contained a high frequency of immunoregulatory sequences with a range of 19.1 to 37.01 among 6 Hp strains, including Hp SS1 [7]. The overall genome sequence of Hp SS1 is very similar to Hp PMSS1 except for intragenomic rearrangement and variation in copy numbers of *cagA* and insertion sequences [34]. Several factors possibly contribute to this discrepancy between our study and published reports. First, Hh-induced intestinal pathology in RAG2 mice is driven by innate immune responses, whereas the results based on the data from humans and female C57BL/6 mice were mediated by a combination of innate and adaptive immune responses. Second, the *S. typhimurium*- or DSS-induced colitis may differ in the interplay between bacterial factors and Hh-induced typhlocolitis in RAG2 mice. There is likely to be cross reactivity between the unique antigenic proteins shared by Hh and Hp, which may be absent between Hp and *S. typhimurium*. This premise is supported by the fact that Hp SS1 shares 385 unique orthologs with Hh 3B1, but only has 61 unique orthologs with *S. typhimurium* LT2 (unpublished data). These cross-reactive antigens between Hp and Hh could potentially enhance Hh-induced proinflammatory immune responses in some circumstances [27]. Finally, pathogenicity among Hp strains is highly variable due to the divergence of virulence determinants encoded in their genomes [35]. Since it has been convincingly established in humans and rodent models that chronic Hp infection elicits proinflammatory Th1/Th17 immune responses that lead to various types of gastric diseases in a subset of infected populations, it is plausible that co-infection with Hp strains may promote IBD in a subset of IBD patients, particularly those that are colonized with selected EHS [36].

Interestingly, co-infection with Hp promoted Hh-induced intestinal pathology in the colon of RAG2 males but not in the cecum where Hh colonization levels (~10^7^) were higher compared to the colon (~10^6^). Comparable severity of cecal pathology between Hh+Hp and mono-Hh mice was in agreement with the finding that co-infection with Hp did not significantly enhance cecal transcription of key ILCs 1/3-associated genes including *Ifnγ*, *Tnfα*, *Il-1β*, and *Il-17A* at 21 WPI. The different impacts of Hp coinfection on Hh-induced cecal versus colonic lesions in RAG2 males may be due to the different roles of proinflammatory cytokines such as Il-17A. Morris et al. reported that neutralization of Il17A aggravated cecal but not colonic pathology in Hh-infected, anti-Il10R-treated C57BL/6 mice, indicating that there was a disease-protective role for Il-17A only in the cecum [37]. Anatomically, the small intestine and the colon are connected through the ampulla ceci and cecocolic junction [38]. Given that Hp does not colonize the lower bowel, it is reasonable to speculate that the majority of Hp or Hp antigens, which traffic from the stomach to the colon through the cecocolic junction, likely bypass the main body of the cecum, which may in part explain why the co-infection with Hp promoted Hh-induced colonic, but not cecal lesions.

In conclusion, we demonstrated that co-infection with Hp promoted Hh-induced colonic carcinogenesis in male RAG2 mice despite the lack of overt Hp gastric pathology. This promotion was coupled with upregulation of a select set of inflammatory cytokines, which was presumably driven via enhancement of colonic ILC1 and ILC3 responses, further highlighting the key roles of these proinflammatory cytokines in the development of CAC. It has been documented that EHS are present in diarrheic patients and have pathogenic potential in the development of IBD in humans [10,39]. Our data suggest that concurrent infection with Hp and selected EHS or bacterial species sharing significant orthologs with Hp could increase a risk of IBD and colonic cancer in a subset of male patients.

## 4. Materials and Methods

### 4.1. Bacterial Strains and Experimental Infections

Mice were maintained in a facility accredited by the Association for the Assessment and Accreditation of Laboratory Animal Care. The animal protocols used in this work were evaluated and approved by the Massachusetts Institute of Technology Committee on Animal Care and Use in accordance with the Guide for the Care and Use of Laboratory Animals (The Research Code # 0912-093-15, 7 September 2012). Five-week-old 129S6/SvEvTac-*Rag2^tm1Fwa^* (RAG2) mice of both sexes, which were originally obtained from Taconic Biosciences (Rensselaer, NY, USA), were maintained and produced at the MIT breeding facility and then housed in groups of five by sex in polycarbonate microisolator cages on hardwood bedding (PharmaServ, Winnipeg, MB, Canada) under specific pathogen free (SPF) conditions (free of *Helicobacter* spp., *Citrobacter rodentium*, *Salmonella* spp., endoparasites, ectoparasites, and exogenous murine viral pathogens). They were maintained at 70 ± 2°F, 30–70% relative humidity, a 12:12 h light to dark cycle, fed standard rodent chow (Purina Mills, St. Louis, MO, USA) and given water *ad libitum*.

A group of 38 mice (20 males, 18 females) was orally gavaged every other day with 3 doses of 0.2 mL (~2 × 10^8^ organisms per dose) of Hh 3B1 (ATCC 51449); a sub group of Hh-infected mice (*n* = 19) received three doses of Hp strain PMSS1 (~2 × 10^8^ organisms per dose), 2 weeks later [40]. Additionally, 19 age-matched mice (10 males, 9 females) were inoculated with Hp alone; 16 sham control mice (10 males, 6 females) were dosed with vehicle (Figure 1). The individual groups were defined as follows: (1) mono-Hh for Hh alone; (2) mono-Hp for Hp alone; (3) Hh+Hp for dual infection with Hh and Hp.

### 4.2. Necropsy and Histopathology

At 10 and 21 weeks post inoculation with Hh (WPI), 8 mice from the control group (5 males, 3 females), 9–10 mice from both mono-Hh and Hh+Hp groups (5 males and 4 females) were euthanized with CO_2_. Stomach samples from the lesser curvature extending from the squamous forestomach through the duodenum were collected and processed as described previously [41].

The cecum, including the ileo–ceco–colic junction and colon (proximal, mid and distal), were collected for routine histological processing and sectioning. Haematoxylin and eosin-stained sections of the intestine were scored for inflammation, edema, epithelial defects, crypt atrophy, hyperplasia, and dysplasia by a boarded veterinary pathologist (SM) who was blinded to sample identity as previously described [12]. An intestinal histologic activity index (HAI) also termed as typhlitis index and colitis index for cecum and colon, respectively, was generated by combining scores for all sub-categorical lesional scores for a particular intestinal segment.

### 4.3. Quantitative PCR for Hp PMSS1 and Hh

To quantify colonization levels of cecal and colonic Hh3B1 and gastric Hp PMSS1, a real-time quantitative PCR assay (qPCR) was utilized. A standard curve was generated using serial 10-fold dilutions of the respective helicobacter genomic DNA representing 1 × 10^6^ to 10 genome copies. Primers and probes for quantifying Hp and Hh were previously described [16]. All qPCR assays were performed in the 7500 Fast Real-Time PCR system (ThermoFisher Scientific, Waltham, MA, USA). Genome copy numbers of the Hp or Hh were expressed per microgram of murine chromosomal DNA which was measured by qPCR using a mammalian 18S rRNA gene-based primer and probe mixture (Life Technology) as described previously [16].

### 4.4. qPCR Analyses of Intestinal Cytokines

Total RNA from murine cecal and colonic tissues was prepared using Trizol Reagents following the supplier’s instructions (ThermoFisher Scientific. Waltham, MA, USA). cDNA from tissue mRNA (2 µg) was reverse-transcribed using the High Capacity cDNA Archive kit following the supplier’s instructions (ThermoFisher Scientific, Waltham, MA, USA). Using the 7500 Fast Real-Time PCR System, mRNA expression of murine genes involved in innate immunity and oncogenesis, including interferon-gamma (*Ifnγ)*, tumor necrosis factor-alpha (*Tnf-α*), and interlukin-1beta (*Il-1β)*, *Il-6*, *Il-10*, *Il-17A*, *Il-17F*, *Il-18*, *Il-22*, *Il-23*, *iNos*, and *regIIIγ*, were measured using primers and probes from Life Technologies. All the target genes were normalized to the endogenous control glyceraldehyde-3-phosphate dehydrogenase (*Gapdh*) mRNA, and expressed as fold change in reference to sham-dosed control mice using the Comparative C_T_ method (Applied Biosystems User Bulletin no. 2).

### 4.5. Statistics

Intestinal HAI scores were compared across groups by the Kruskal–Wallis one-way analysis of variance with Dunn’s post-test, and between groups by the two-tailed Mann–Whitney U-test using the Prism software Package (Graphpad, San Diego, CA, USA). Data on the colonization levels of *Helicobacter* species and cytokine mRNA levels in the tissues were analyzed using the two-tailed Student’s *t* test. Values of *p* < 0.05 were considered significant.

## Supplementary Materials

Supplementary materials can be found at https://www.mdpi.com/1422-0067/21/23/8886/s1. Figure S1, Male gastric histology; Figure S2, Male cecal pathology; Figure S3, Female colonic pathology; Figure S4, Colonic gene expression; Figure S5, Cecal gene expression.

## Abbreviations

CRC	Colorectal cancer
EHS	Enterohepatic *Helicobacter*
HAI	Histologic activity index
Hh	*Helicobacter hepaticus*
Hh+Hp	Coinfection with Hh and Hp
Hp	*Helicobacter pylori*
IBD	Inflammatory bowel disease
Ifnγ	Interferon gamma
IL	Interleukin
ILC	Innate lymphoid cells
iNos	Inducible nitric oxide
NK	Natural killer cells
PCR	Polymerase chain reaction
RegIIIγ	Regenerating islet-derived protein 3 gamma
RAG2	129S6/SvEvTac-*Rag2^tm1Fwa^*
Rag2	Recombination activating gene 2
Tnfα	Tumor Necrosis factor alpha
WPI	Weeks post-Hh infection
