# Supplementary material for: Male-Dependent Promotion of Colitis in 129 Rag2−/− Mice Co-Infected with Helicobacter pylori and Helicobacter hepaticus
Source: Int J Mol Sci. 2020 Nov 24;21(23):8886. doi: 10.3390/ijms21238886 (PMC7727654; doi:10.3390/ijms21238886)
Supplement: Supplementary file 1 [file ijms-21-08886-s001.pdf]

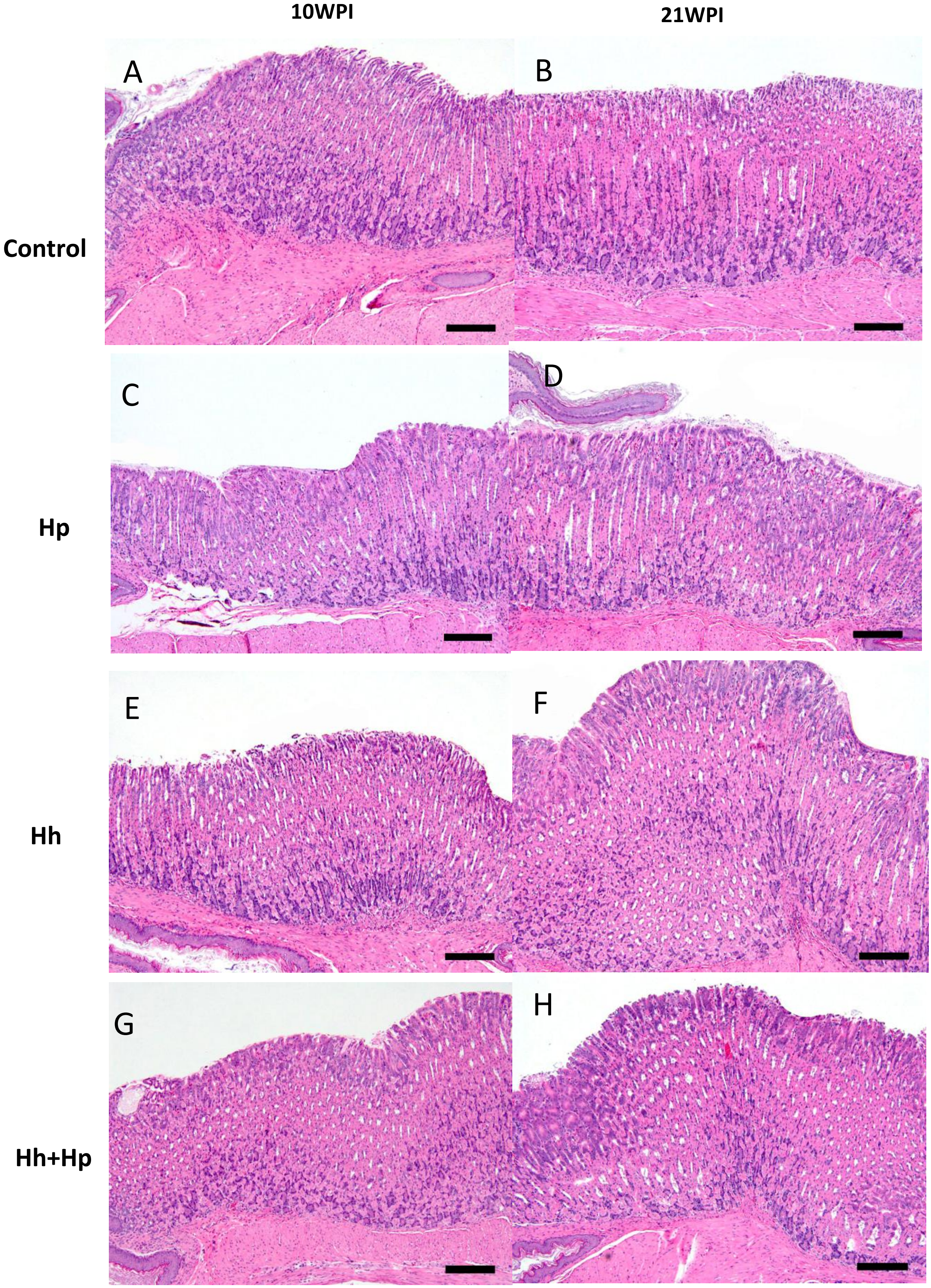

Figure S1. Gastric pathology

Figure S1. Representative H&E images of stomach from male mice of different groups at 10 WPI and 21WPI. A: Uninfected male at 10 WPI. B: uninfected male 21 WPI. C: mono-Hp male at 10 WPI. D: mono-Hp at 21 WPI. E: mono-Hh at10 WPI. F: mono-Hh male at 21 WPI. G: Hh+Hp male at 10 WPI. G: Hh+Hp male at 21WPI. All sections showing normal gastric corpus and adjacent squamous –columnar junction with no discernible inflammation or oxyntic loss. BAR, all images: 150μM. Gastric pathology in females were similar to the corresponding groups of males.

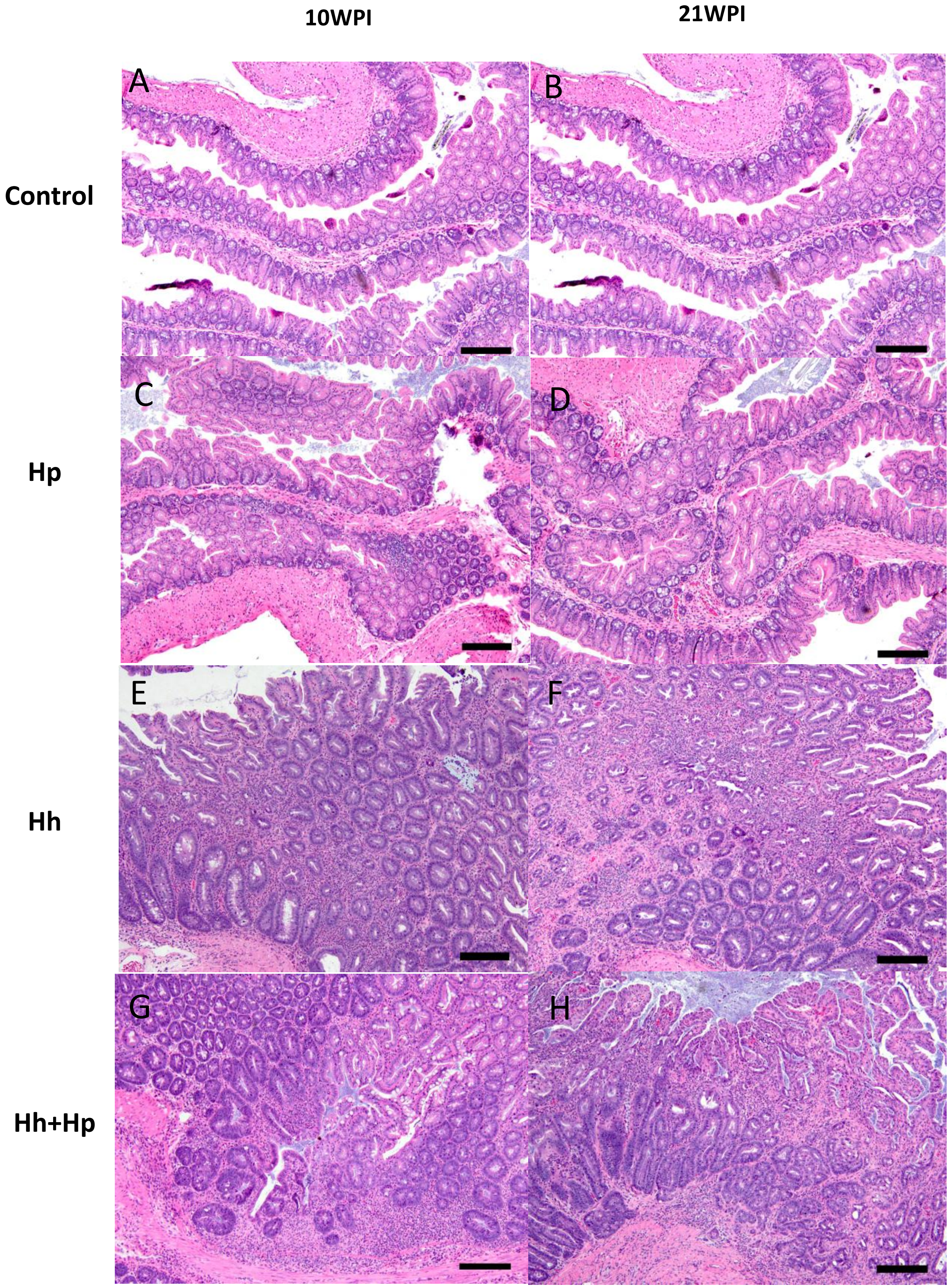

Figure S2. Cecal Histology

Figure S2: Representative H&E images of cecum from male mice of different groups at 10 and 21WPI. A: Uninfected at 10 WPI. B: uninfected at 21 WPI. C: mono-Hp at 10 WPI. D: mono-Hp at 21 WPI. E: mono-Hh at 10wpi. F: mono-Hh at 21 WPI. G: Hh+Hp at 10 WPI. H: Hh+Hp at 21 WPI. Panels A-D showing none to patchy lamina proprial inflammatory aggregates comparable across the four groups. Panel E showing severe diffuse mucosal and sub-mucosal inflammation (chiefly neutrophils and macrophages) with mild edema, glandular hyperplasia and mild dysplasia. Panel F showing severe mucosal and sub-mucosal inflammation, fibrosis, papillary hyperplasia and high grade dysplastic glandular proliferation with invasion and partial effacement of the muscularis mucosa. Panel G showing severe mucosa inflammation, mild erosions, crypt loss, prominent epithelial hyperplasia and high grade glandular dysplasia. Panel H showing severe inflammation and villo-papillary adenocarcinomatous epithelial proliferation with early invasion into the submucosa. BAR, all images: 150µM. The pathological features in the ceca of females were similar to the corresponding groups of males.

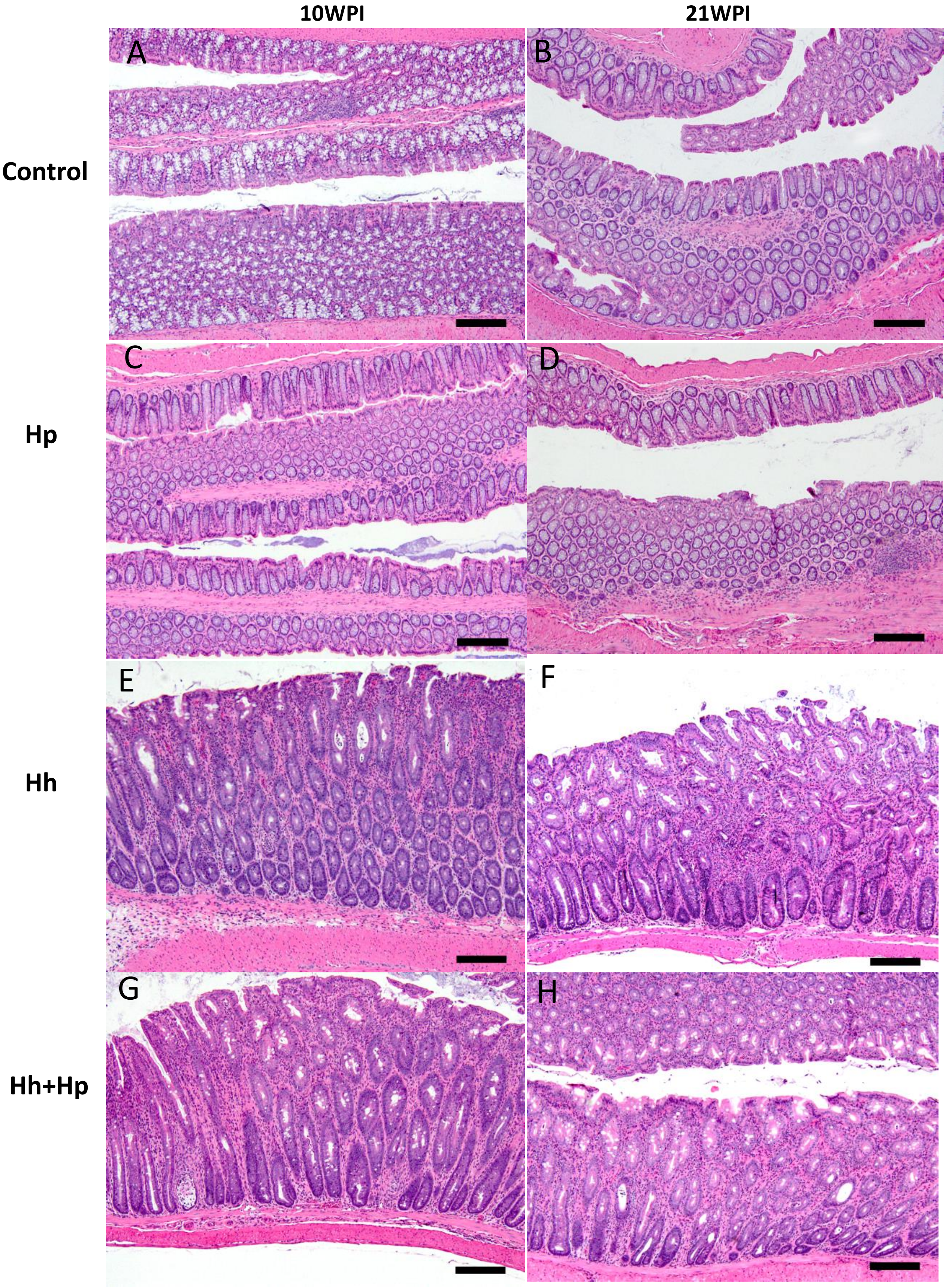

Figure S3. Female colonic Histology

Figure S3: Representative H&E images of colon from female mice of different groups at 10 WPI and 21 WPI. A: uninfected female at 10 WPI. B: uninfected female at 21 WPI. C: mono-Hp female at 10 WPI. D: mono-Hp female 21 WPI. E: mono-Hh female at 10 WPI. F: mono-Hh female at 21 WPI. G: Hh+Hp female at 10 WPI. H: Hh+Hp female at 21WPI. Panels A-D depict normal colons with no significant inflammation. Panels E-H that show moderate to severe inflammation, epithelial defects, moderate epithelial hyperplasia and mild to moderate dysplasia in mono-Hh groups at both time points. BAR, all images: 150µM.

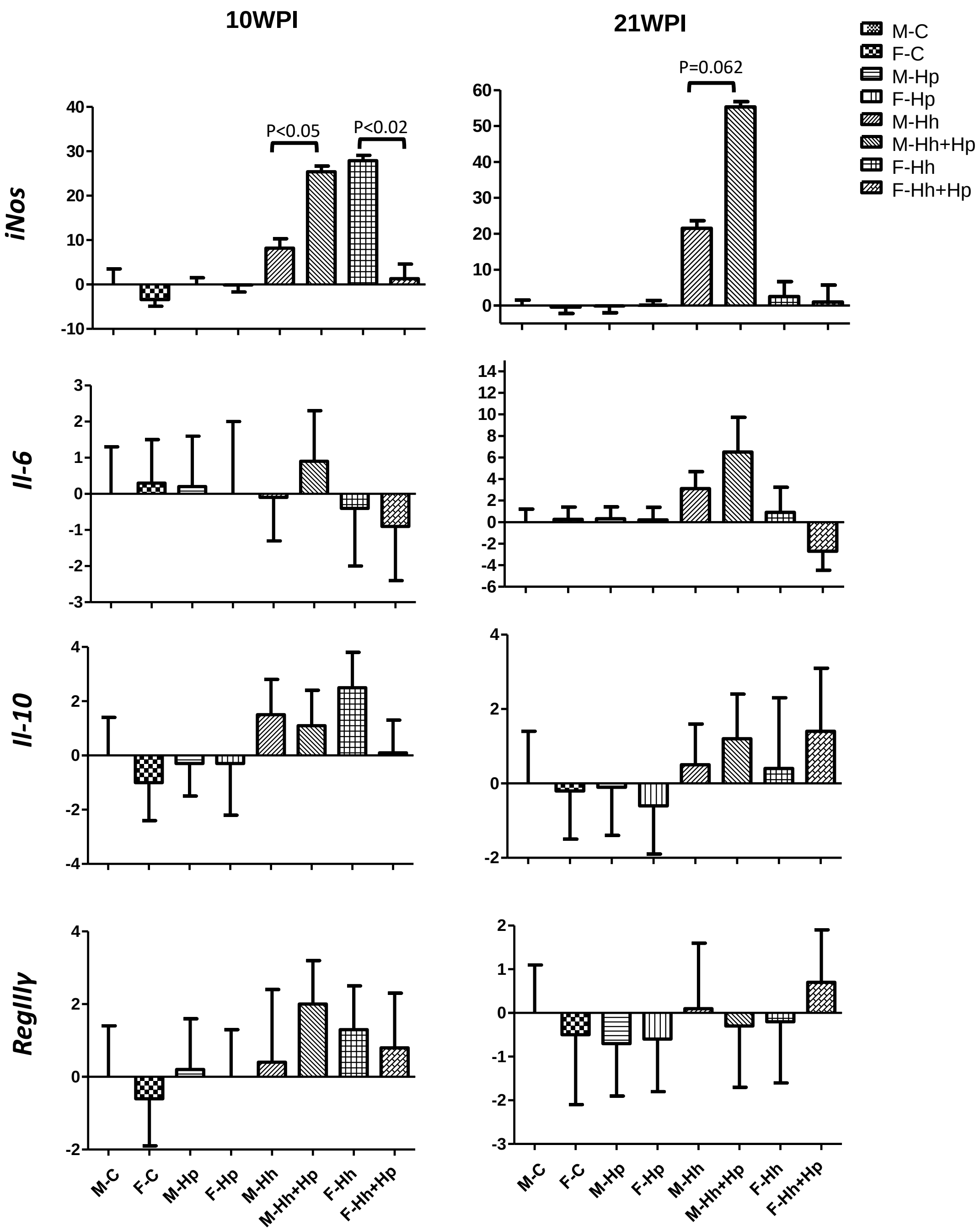
10WPI
21WPI
M-C
F-C
M-Hp
F-Hp
M-Hh
M-Hh+Hp
F-Hh
F-Hh+Hp
iNos
Il-6
Il-10
RegIIIγ
P<0.05
P<0.02
P=0.062

Figure S4

Figure S4. Co-infection with Hp in males did not increase mRNA levels of genes being implicated in colonic carcinogenesis when compared to mono-Hh males. Total RNA prepared from colonic tissues of mice infected or sham-dosed were evaluated by qPCR for expression levels of mRNA for select cytokines, which then were normalized to the expression of the house-keeping gene *Gapdh*. The Y axis represents the mean fold change (± standard deviation) of the mRNA levels in reference to uninfected male controls.

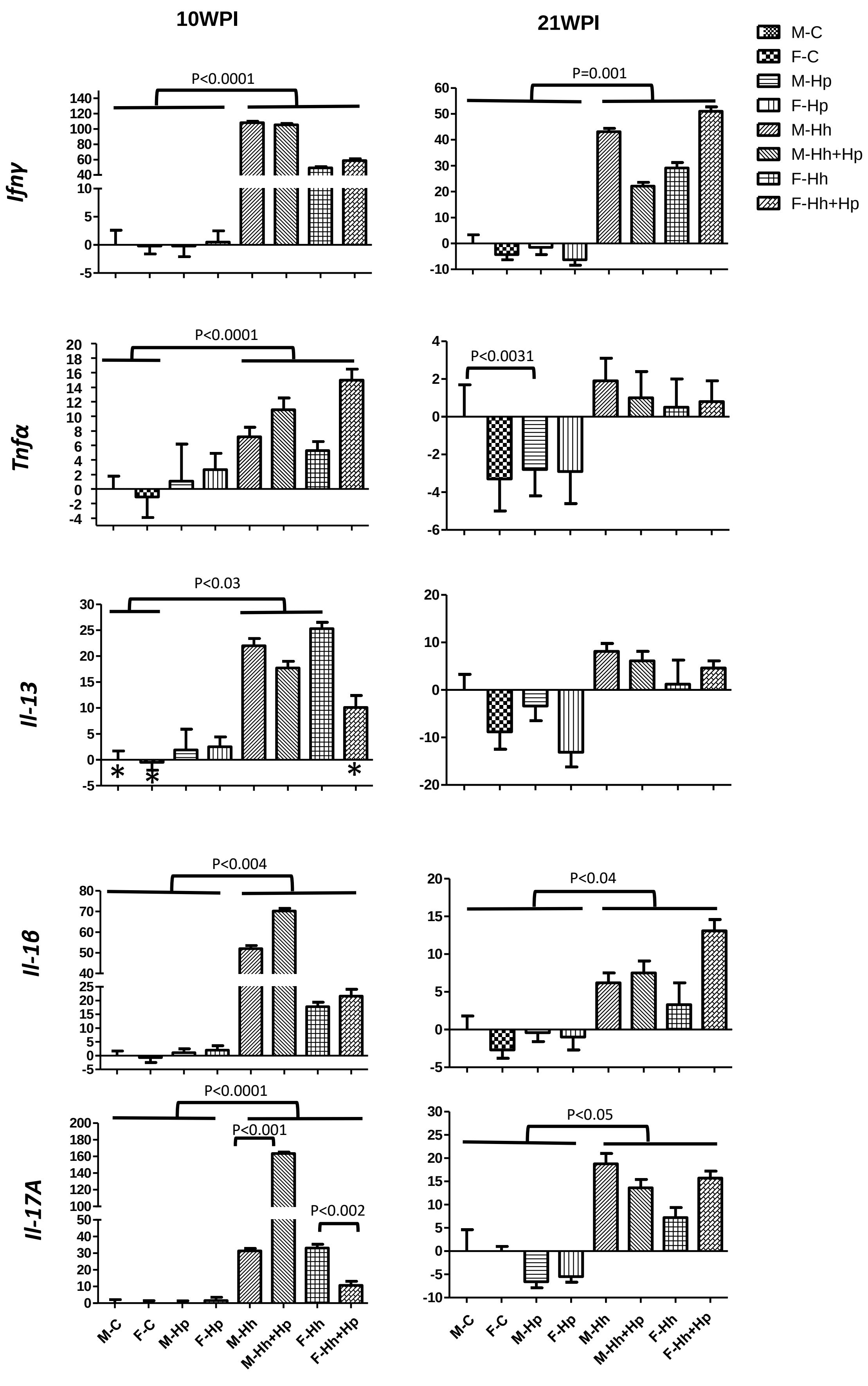

Figure S5. Cecal gene expression
(continued on the next page)

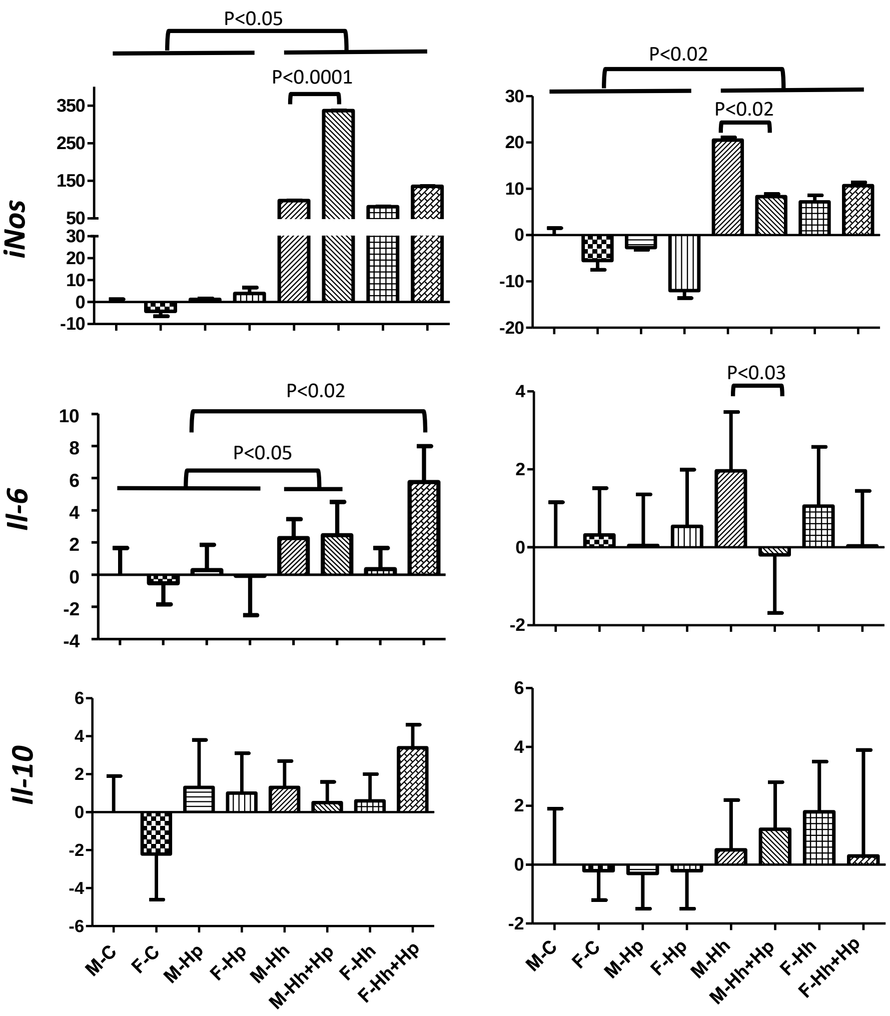

Figure S5. Cecal gene expression

Figure S5. mRNA levels of cecal ILC1s and ILC3s-associated genes were not enhanced in Hh+Hp males compared to mono-Hh counterparts. Total RNA prepared from colonic tissues of mice infected or sham-dosed were evaluated by qPCR for expression levels of mRNA for select cytokines, which then were normalized to the expression of the house-keeping gene *Gapdh*. The Y axis represents the mean fold change (± standard deviation) of the mRNA levels in reference to uninfected male controls.
